# Assessment of health impacts in retired antisera-producing horses: Blood biochemistry and serum amyloid A analysis

**DOI:** 10.14202/vetworld.2024.2136-2143

**Published:** 2024-09-20

**Authors:** Dinar Arifianto, Anita Esfandiari, I Wayan Teguh Wibawan, Amrozi Amrozi, Maharani Maharani, Darsono Darsono, Hirawan Setiadi, Agus Setiyono

**Affiliations:** 1 School of Veterinary Medicine and Biomedical Science, IPB University, Jl. Agatis, Kampus IPB Dramaga 16680, Bogor, Indonesia; 2 Faculty of Veterinary Medicine, Universitas Gadjah Mada, Jl. Fauna No. 2 Karangmalang, Catur Tunggal, Yogyakarta, Indonesia; 3 Bio Farma (Persero), Jl. Pasteur No. 28, Bandung 40161, West Java, Indonesia

**Keywords:** antisera, blood biochemistry, horse, hyperimmune, serum amyloid A

## Abstract

**Background and Aim:**

Horses used for antisera production are repeatedly hyperimmunized to produce high levels of specific antibodies. This prolonged process can lead to various health issues, including amyloidosis, which involves the accumulation of amyloid proteins in organs and tissues, potentially causing organ dysfunction and failure. These horses are often retired when they no longer produce adequate antibody levels. This study aimed to evaluate the impact of prolonged antisera production on the health of retired horses by examining their blood biochemistry and serum amyloid A (SAA) levels, which are indicators of systemic inflammation and organ damage.

**Materials and Methods:**

Blood samples were collected from 12 horses for this study. Nine horses were retired antisera-producing horses that had been discontinued for 2 years, while three healthy non-antisera-producing horses were used as controls. These twelve horses were divided into four groups based on the duration of their active period as antisera producers (never been used, 2–3 years, 4–5 years, and 6–7 years). We measured key blood biochemistry parameters and SAA levels to evaluate the health status of the horses.

**Results:**

Total protein, fibrinogen, and globulin levels were elevated, whereas other parameters remained normal. The findings indicate that despite normal SAA levels, the horses exhibited signs of ongoing health issues related to their previous use in antisera production, such as increased total plasma protein, fibrinogen, and globulin levels, as well as the presence of amyloid deposits in vital organs such as the liver and kidneys, as observed in post-mortem examinations.

**Conclusion:**

Despite normal SAA levels, retired antisera-producing horses showed elevated total protein, fibrinogen, and globulin levels, indicating ongoing health issues.

## Introduction

Horse is an important animal in antisera production [1, 2]. Horse antisera obtained through the polyclonal antibody technique is used as passive human immunization in various cases of bacterial, viral, venom, and biological toxin infections [3]. Antisera production animals are hyperimmunized with certain immunogens to obtain specific immunoglobulins. After several purification and fractionation processes, immunoglobulins are used for human medical purposes [4]. Prolonged hyperimmunization during horse antisera production leads to the deterioration of horse health quality. Amyloidosis is the most common side effect in horses [5]. Amyloidosis is a group of diseases caused by protein misfolding that results in deposits of amyloid fibrils in extracellular tissues, leading to organ damage and dysfunction [6].

Equine-derived antisera production is still widely used because of its lower production costs and shorter production time compared to monoclonal antibody techniques [7]. Therefore, it is essential to ensure that the welfare and health status of horses are well conserved. Amyloid deposits can lead to bleeding, failure, and organ rupture, making biopsy procedures the gold-standard diagnosis even more risky. A non-invasive diagnostic method is needed to identify organ damage and potential health problems. Blood biochemistry and serum amyloid A (SAA) tests can be performed in animals with suspected amyloidosis [1]. These tests can provide an overview of systemic inflammation and organ function [8].

Antisera-producing horses are often retired when they no longer produce adequate antibody levels. Assessing the horse’s health must ensure that the horse can retire prosperously. Prior biochemical studies of the blood of antisera-producing horses have focused on examining horses that are still actively used as antisera producers. However, there is a lack of information on the health evaluation of retired horses.

This study aimed to evaluate the health impacts of retired antisera-producing horses by assessing their blood biochemistry and SAA levels. The results of this investigation are intended to improve the welfare of antisera-producing horses and guide future antisera production practices.

## Materials and Methods

### Ethical approval

The Ethics Committee of the School of Veterinary Medicine and Biomedical Science, IPB University, approved all procedures in this research including the euthanasia process (certificate number: 028/KEH/SKE/IX/2022). Blood samples were collected from the horses by a trained person as per the standard sample collection procedure without giving any unnecessary stress. Euthanasia was performed using the pentobarbital method.

### Study period and location

The study was conducted from December 2022 to July 2023 at the IPB Equestrian Center, School of Veterinary Medicine and Biomedical Science, IPB University Bogor, Indonesia.

### Experimental design

This study involved 12 horses, including nine retired antisera-producing horses and three healthy non-antisera-producing horses that were used as controls. The retired antisera-producing horses were acquired from an antisera company and have been adopted by the university stable. They have been retired from hyperimmunization for 2 years. The average weight of the horses was 250–350 kg. The types of immunogens given to these horses included Tetanus, Diphtheria, Naja, Bungarus, and Agkistrodon-Bungarus-Naja (poly-specific immunogen) (Table-1). These 12 horses were divided into four groups (three horses each) based on the duration of their active period as antisera producers. Group I consisted of horses used for antisera production for 2–3 years; group II for antisera production for 4–5 years; group III for antisera production for 6–7 years; and the control group. Gender and immunogen type were not considered in the grouping process.

**Table-1:** Data of twelve horses used in the study. The horses were divided into four groups based on the duration of exposure (length of active period) as antisera producers.

Horse data	Control (n = 3)	Group I (n = 3)	Group II (n = 3)	Group III (n = 3)
Age (years)				
Mean	10.3	13	12	14.3
Median	8	12	12	13
Age range	8–15	12–15	9–15	13–17
Gender				
Male	0	2	3	2
Female	3	1	0	1
The type of immunogen				
Tetanus	0	1	0	1
Diphtheria	0	0	1	0
Naja	0	0	0	1
Bungarus	0	0	1	1
Agkistrodon-Bungarus-Naja	0	2	1	0

Control group (never been used as antisera producers), Group I (length of active period 2–3 years), Group II (length of active period 4–5 years), Group III (length of active period 6–7 years)

Nine milliliters of blood samples were taken from the jugular vein and placed into lithium heparin, *tripotassium ethylenediamine tetraacetic acid* (K_3_EDTA), and serum separator tubes. Samples for blood biochemical examination were taken once per horse, whereas serum samples for SAA analysis were collected four times per horse (days 1, 4, 7, and 30). Blood biochemistry parameters were tested on the same day as sample collection, and SAA was tested on the following day. Samples were stored at −20°C before testing. Blood biochemistry examination included total plasma protein, albumin, globulin, fibrinogen, aspartate aminotransferase (AST), gamma-glutamyl transferase (GGT), total bilirubin (TBIL), creatine kinase (CK), blood urea nitrogen (BUN), and creatinine (CRE). Fibrinogen was measured using heat precipitation. Other biochemical tests were examined with Abaxis VetScan VS2™ (Abaxis Inc. Union City, California, USA) automatic analyzer using equine profile plus rotor. The VetScan VS2 Analyzer can calibrate independently. The bar code on the reagent rotor contains the information required to calibrate the rotor when run. Each reagent bead used in the rotor was calibrated according to the reference method. SAA levels were analyzed using enzyme-linked immunosorbent assay with a horse SAA kit (FineTest™, Wuhan Fine Biotech Co., Ltd. Hubei, China). Amyloidosis that develops due to hyperimmunization potentially affects various organs, and this condition requires a non-invasive diagnostic method to determine organ function through blood biochemical examination. SAA is an important acute-phase protein for horses; on the other hand, it is a precursor of equine systemic amyloidosis. The examination of SAA is aimed to reveal the timing and pattern of amyloidosis in antisera-producing horses.

### Statistical analysis

Statistical analysis begins with the normality and homogeneity of variances test; if it meets the conditions, then the two-way analysis of variance test was carried out; if it does not meet the conditions, then the non-parametric tests (Kruskal-Walis and Mann-Whitney) were used. All statistical data were processed using the Statistical Package for the Social Sciences version 25 Software (IBM Corp., NY, USA). One horse from group III was euthanized humanely due to severe blindness caused by bilateral uveitis and lower body condition score.

## Results

### Protein components

#### Total plasma protein

**Figure-1:**
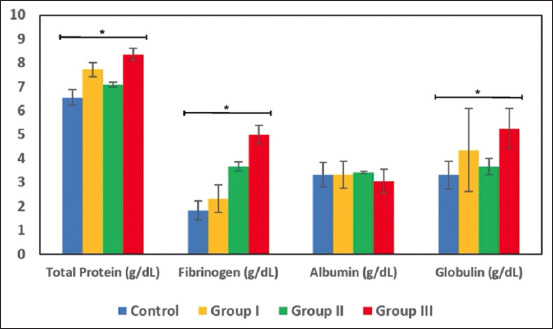
Graphic illustrates measurement of total protein, fibrinogen, albumin, and globulin levels in hyperimmune horses used for antisera production. Asterisks (*) indicate significant differences according to the Kruskal–Wallis test (p < 0.05). Total plasma protein, fibrinogen, and globulin levels were significantly higher in Group III compared with the control, indicating a prolonged effect of antisera production on protein component levels.

Kruskal–Wallis analysis showed significant differences in total plasma protein, fibrinogen, and globulin levels (p < 0.05). Mann–Whitney test analysis was performed to determine which groups differed significantly. The total plasma protein values of the control and group I were significantly different. Other significant differences were found between groups I and II and between groups II and III. The total plasma protein values of group III were the highest among all groups and were above the normal limits. The detailed results are presented in Table-2 and illustrated in Figure-1. Statistical analysis found that horses hyperimmunized for 2–3 years and 6–7 years had high total plasma protein levels after 2 years of retirement. Significant elevation in plasma protein levels in group III indicates a physiological response or serious health problem because of prolonged hyperimmunization.

**Table-2:** Protein component levels of retired antisera-producing horses. Significant differences were found in the total plasma protein, fibrinogen, and albumin levels. However, there were no significant differences in albumin levels.

Parameters	Mean ± Standard deviation				p-value	Normal range Southwood [9]

	Control	Group I (2–3-year production)	Group II (4–5-year production)	Group III (6–7-year production)		
Total protein level (g/dL)	6.56 ± 0.32^a^	7.73 ± 0.4^b^	7.1 ± 0.51^a^	8.36 ± 0.58^c^	0.025*	5.7–8.0
Fibrinogen (g/dL)	1.83 ± 0.29^a^	2.33 ± 0.57^ab^	3.67 ± 0.57^b^	5.0 ± 1.73^b^	0.036*	1.5–3.75
Albumin (g/dL)	3.33 ± 0.1	3.33 ± 0.2	3.43 ± 0.05	3.06 ± 0.35	0.272	2.5–4.2
Globulin (g/dL)	3.33 ± 0.26^a^	4.36 ± 0.4^b^	3.67 ± 0.49^ab^	5.26 ± 0.83^c^	0.031*	2.7–5.0

The asterisk sign (*) indicates a significant difference at the Kruskal–Wallis test level (p < 0.05). Similar superscript letters within the same row indicate no significant difference at the Mann–Whitney test level, while different superscript letters indicate a significant difference at the Mann–Whitney test level

#### Fibrinogen

Mann–Whitney analysis revealed significant differences in fibrinogen levels between the control and group II and between the control and group III. Group III showed fibrinogen levels above the normal limit. Data are presented in Table-2. These results suggest that hyperimmunization for more than 4 years affects fibrinogen levels despite 2 years of horse retirement. Figure-1 illustrates that the highest fibrinogen value was observed in group III. The significant increase in fibrinogen levels in group III suggests a possible inflammatory response due to prolonged antisera production.

#### Albumin

There were no significant differences in albumin levels between the control and the other groups (p > 0.05). Table-2 shows that the highest albumin levels were observed in group II. In contrast, the lowest levels were observed in group III. However, all albumin levels were within the normal range. Hyperimmune horses with normal albumin concentrations may still have good hepatic function.

#### Globulin

Significant differences in globulin levels were found between control and group I, control and group III, group I and group III, and group II and group III. Data are presented in Table-2. The highest mean globulin level was observed in group III (Figure-1), which was above the normal range. The significant differences in total plasma protein, fibrinogen, and globulin levels indicate that these parameters are affected by the duration of antisera production in horses, suggesting that prolonged production may lead to increased protein component levels.

### SAA

**Figure-2:**
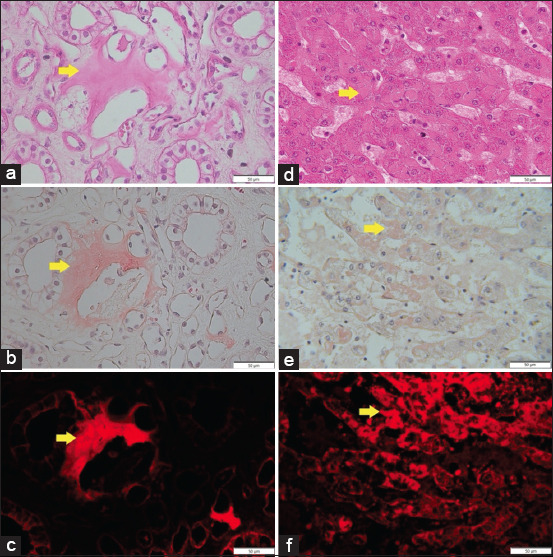
(a) Eosinophilic extracellular matrix around renal tubules (HE staining); (b) Congo red staining of kidney tissue observed under light microscope; (c) Congo red staining of kidney tissue under fluorescence microscope; (d) Eosinophilic extracellular matrix around hepatocytes (HE staining); (e) Congo red staining of liver observed under light microscope; (f) Congo red staining of liver under a fluorescence microscope. Yellow arrows indicate amyloid deposits. 400× magnification.

Statistical analysis of SAA concentration showed a significant difference between groups I and II on all sampling days (p < 0.05). However, the average of all SAA examination results was within normal limits. This indicates that horses did not have any acute inflammation occurring, and the SAA was not produced at high levels despite the presence of amyloid deposits on tissue examination (Figure-2). Observations from day 1 to day 30 also did not reveal significant fluctuations in SAA levels. These data suggested no correlation between SAA levels and amyloid concentrations in tissues when the source of inflammation (hyperimmunization) was stopped for 2 years.

### Enzymes and other biochemical components

There were no significant differences in AST, GGT, TBIL, CK, BUN, and CRE levels between the groups (p > 0.05). However, Table-3 shows that the AST and TBIL levels of group II were above reference range as described by Orsini and Divers [10]. An increase in AST and TBIL indicates potential health problems related to decreased organ function in retired antisera-producing horses.

**Table-3:** Levels of AST, GGT, TBIL, CK, BUN, and CRE in retired antisera-producing horses. The high levels of AST and TBIL in group II and group III, respectively, indicate potential health problems.

Parameters	Mean ± standard deviation				p-value	Reference value [10]

	Control	Group I (2–3 years production)	Group II (4–5 years production)	Group III (6-–7 years production)		
AST (U/L)	256.33 ± 45.00	307.33 ± 86.73	367.33 ± 107.22	305.67 ± 101.79	0.45	102–350
GGT (U/L)	19.33 ± 10.06	25 ± 13.89	16.67 ± 2.51	27 ± 7	0.36	10–40
TBIL (mg/dL)	0.93 ± 0.45	2.06 ± 0.11	1.63 ± 0.97	2.83 ± 1.59	0.19	0.5–2.3
CK (U/L)	210.33 ± 81.24	131.67 ± 14.15	210.33 ± 50.52	131.67 ± 17.67	0.97	90–270
BUN (mg/dL)	13.33 ± 0.57	19.0 ± 4.58	22.33 ± 1.15	17.33 ± 4.5	0.90	8–27
CRE (mg/dL)	1.43 ± 0.15	1.67 ± 0.2	1.83 ± 0.2	2.0 ± 0.52	0.14	0.6–18

Statistical analysis using Kruskal-Wallis test indicates no significance difference so that Mann–-Whitney analysis was not employed

### Pathological findings

**Figure-3:**
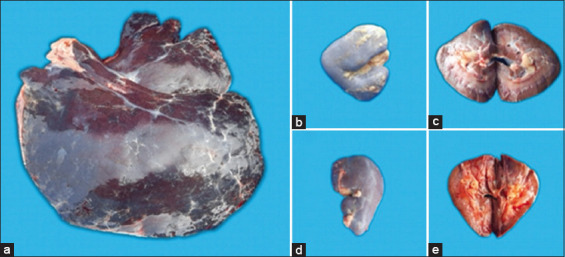
(a) Organ examination (euthanized horses from group III) showed hepatomegaly in the liver. (b and c) The right kidney. (d and e) the left kidney did not undergo macroscopic changes.

Hepatomegaly and darker discoloration occured in the liver of euthanized horse (Figure-3). Histopathology examination showed an eosinophilic hyaline extracellular matrix surrounding hepatocytes and sinusoids. Amyloid dispersed around hepatocytes appeared pink or salmon red when observed using a light microscope (Olympus BX43, Japan) at 400× magnification. Fluorescence microscope observation using a Euromex bScope trinocular with EX540 filter at a wavelength of 528–552 nm (Euromex, Holland) showed that amyloid disposition appeared bright red with dark red normal cells and a black background (Figure-2). In addition, positive amyloid deposition was found in the renal tubules (Figure-2) despite the absence of abnormalities on gross anatomy examination (Figure-3).

## Discussion

After being rested for 2 years, horses with hyperimmunization periods of 2–3 years and 6–7 years showed high total plasma protein levels. Hyperproteinemia that occurs concurrently with hyperfibrinogenemia and hyperglobulinemia should be considered as a sign of inflammation or a potential health problem. These results are in accordance with those of a previous study by Moreira *et al*. [5], which reported high levels of total protein, fibrinogen, and globulin as indicators that can be associated with hepatic amyloidosis. Total plasma protein can be used to determine the physiological and health status of horses [11]. The major fractions of plasma proteins are fibrinogen, albumin, and globulin. Elevated fibrinogen and globulin levels increase total plasma protein levels [12]. Prolonged and continuous hyperimmunization caused a significant increase in globulin levels, even after the horses had been retired for 2 years. Hyperimmunization for 6–7 years increases globulin levels. Horses with liver disease can present a high level of globulin levels up to 48% [13]. Hyperglobulinemia in antisera-producing horses is derived from high gamma-globulin levels [14]. It mainly comprises immunoglobulins, which are produced by B lymphocytes and plasma cells. Chronic antigen stimulation increases the immunoglobulin concentration, followed by an elevation in globulins and total plasma protein [15].

Hyperimmunization for >4 years affected fibrinogen levels even if the horse had been retired for 2 years, and the highest fibrinogen values were found in the group with a length of active period of 6–7 years (group III). Elevated fibrinogen levels have been reported in one case of hepatic amyloidosis confirmed by ultrasound examination and liver biopsy [5]. High fibrinogen concentrations can be an important indicator of inflammation. However, this phenomenon can be observed in dehydrated horses [16]. Hyperproteinemia accompanied by hyperglobulinemia, hyperfibrinogenemia, and normal albumin levels indicate ongoing inflammation [17]. Fibrinogen is a plasma-soluble glycoprotein synthesized in the liver. Despite the uncommon incidence of hypofibrinogenemia in severe hepatopathy, dysfibrinogenemia, which involves abnormal production of fibrinogen molecules, is much more prevalent [18]. Fibrinogen is converted into fibrin in the presence of thrombin, which is important in the blood clotting. In addition, it binds to cell surface integrins (CD11/CD18) found in phagocytic cells, which initiate phagocytosis, degranulation, and antibody-dependent cytotoxicity. The normal concentration of equine fibrinogen is between 2 and 4 g/dL and can increase up to 1–10 times during an acute phase reaction over 24–72 h [19]. Fibrinogen is one of the best acute-phase proteins in horses besides SAA. Elevated levels of protein components, including fibrinogen, in retired antisera-producing horses, should be considered an indicator of serious health problems [20]. During the 2-year rest period, horses are not exposed to immunogens but still have high protein component levels. Moreira *et al*. [5] reported that equine patients with hepatic amyloidosis treated continuously for 28 days with antibiotics and glucocorticoids were able to improve serum protein and GGT levels.

Hyperimmune horses that had been retired and rested for 2 years showed normal albumin levels, and no significant differences were found between the control and other groups. In an experimental study by Parraga *et al*. [21], hyperimmunization was found to cause hypoalbuminemia. Albumin can also be used as an indicator of hepatic damage, so hyperimmune horses with normal albumin concentrations potentially have good hepatic function. Albumin plays an important role in the regulation of osmotic pressure, the carrier of hormones, fatty acids, metal ions, and drugs [10].

The statistical analysis of AST and GGT values revealed no significant differences between the groups. Although the AST and GGT parameters did not significantly increase, it is important to be aware of the possible liver disorders. Proven by amyloid deposits in the liver of one of the euthanized horses, but not detected through AST and GGT examinations. In addition, kidney function parameters showed that BUN and CRE levels remained within normal limits, despite amyloid deposits in the renal parenchyma. The GGT level in antisera-producing horses changes dramatically when used continuously for 7–8 years [1]. A previous study by Hablovarid *et al*. [22] reported that GGT and TBIL levels in active antisera-producing horses that were used for 9 years remained normal, despite BUN levels showing an increase in horses that were actively used for 3–9 years. When horses reach 10 years of production time, AST, GGT, BUN, and CRE levels increase. The liver has remarkable adaptability, allowing it to continue to function despite a large amount of tissue damage. Clinical symptoms and elevated liver enzyme levels emerge only when liver damage exceeds 70% [22, 23]. GGT is a membrane-bound enzyme associated with the equine biliary cell epithelium [24]. The largest production of GGT occurs in the liver; however, the pancreas, intestines, and kidneys also produce small volumes of GGT. Elevated GGT concentrations are associated with cholestasis or biliary hyperplasia. GGT values are more sensitive for diagnosing equine biliary disorders than ALPs [25]. When amyloid deposits are present in renal blood vessels and tubules, BUN and CRE levels are usually unaffected or at normal levels [26]. Renal amyloidosis affecting a large area of the glomeruli causes proteinuria in humans [27].

This study found that antisera-producing horses that were hyperimmunized for 3–7 years and followed by retirement for 2 years showed normal levels of SAA. However, there was a significant increase in SAA levels between group I (2–3 years of active period) and group II (4–5 years of active period). Significant differences may represent variations in the baseline values of antisera-producing horses within the groups, as demonstrated by identical mean, standard deviation, and significance values in each sampling period. The normal range of equine SAA is below 20 mg/L. Some conditions, such as horse breed and age, may affect the minimum normal threshold value of SAA for each individual [28]. Amyloidosis is a life-threatening condition caused by the accumulation of insoluble extracellular fibril aggregates [29]. Type AA amyloidosis is the most common type found in antisera-producing horses. It is associated with chronic inflammatory processes, neoplastic diseases, or idiopathic [27]. In this type, amyloid deposits are derived from SAA, which is an acute-phase reactant [30]. SAA is an apolipoprotein of high-density lipoprotein classes 2 and 3. It is synthesized primarily in the liver due to the stimulation of proinflammatory cytokines and plays a major role in cholesterol transport and chemoattractants during inflammatory processes [31]. Immunization triggers immune cells to produce proinflammatory cytokines that trigger the hepatic synthesis of the acute phase proteins SAA [20, 32]. SAA levels can increase hundreds of folds from normal levels in acute inflammatory conditions [33]. It will increase at least 6 h from the inflammatory stimulus, and if the inflammation is resolved, it can immediately decrease as early as 12 h [10]. This can be observed in Table-4, which presents significant differences between groups. However, the values were still within normal limits [34]. When hyperimmunization is stopped and the body responds properly, SAA levels gradually decrease to normal. In human patients, cases of AA amyloidosis can be controlled by keeping SAA levels below 10 mg/L [35]. Since the level of SAA in human serum reflects amyloid deposits in tissues, the assessment of SAA levels can be used as a guideline for the treatment of AA amyloidosis. The SAA levels will decrease following the reduction of tissue amyloid deposits [36].

**Table-4:** Serum amyloid A (SAA) levels of each group on days 1, 4, 7, and 30. A significant difference was found between groups I and II on all sampling days (p < 0.05).

Groups	Day 1	Day 4	Day 7	Day 30	Reference value [12]
Control	7.96 ± 1.76^ab^	8.23 ± 1.15^ab^	6.95 ± 1.74^ab^	7.71 ± 3.18^ab^	0–20 mg/L
Group I	5.58 ± 0.33^a^	6.03 ± 0.71^a^	5.39 ± 3.27^a^	6.48 ± 0.44^a^	0–20 mg/L
Group II	9.98 ± 5.91^b^	10.91 ± 5.77^b^	11.10 ± 6.01^b^	10.82 ± 5.52^b^	0–20 mg/L
Group III	9.29 ± 6.18^ab^	9.56 ± 6.53^ab^	10.49 ± 6.82^ab^	10.42 ± 4.83^ab^	0–20 mg/L

Different superscript letters in rows or columns indicate significant differences (error rate of 0.05. p-value (group) < 0.05; p-value (collection day)=0.986

Prolonged hyperimmunization leads to the formation of AA-amyloid deposits in various tissues [27]. The deposition of insoluble amyloid fibrils is aggravated by the inability of enzymes to degrade SAA or the synthesis of abnormal SAA proteins that are resistant to enzyme degradation [14]. SAA isoforms are partially broken down into fragments that have a tendency to form aggregates of amyloid fibrils that can be deposited systemically, especially in the kidneys, liver, and spleen [37]. HE staining alone is not sufficiently accurate to diagnose amyloidosis; therefore, it is necessary to apply Congo red special staining [38]. Cazzaniga *et al*. [39] and Fussell *et al*. [40] revealed that Congo red staining showed fluorescence activity when it binds to amyloid fibrils and is visualized under ultraviolet light. As a fluorochrome, it emits red light fluorescence that can be captured if it has a suitable wavelength (530–585 nm) and light emission of more than 600 nm [41]. In this study, we successfully captured extracellular matrix formation confirmed as amyloid by Congo red staining. The filter used in this study was EX540 with a wavelength of 528–552 nm so that it could visualize the accumulation of tissue amyloid properly (Figure-2). This information is important to note because amyloid deposits in tissues do not necessarily affect blood biochemistry and SAA levels.

## Conclusion

Elevations in protein, fibrinogen, and blood globulin levels may indicate health impacts in antisera-producing horses, and further monitoring of retired horses is necessary. One limitation of this study is the relatively small sample size, which may limit the generalizability of the findings. In addition, the 2-year rest period may not fully mitigate the long-term effects of hyperimmunization. Future studies should investigate the long-term health effects of hyperimmunization beyond 2 years of rest and explore interventions that can mitigate elevated globulin and fibrinogen levels.

## Authors’ Contributions

DA and AS: Conducted the study and drafted and revised the manuscript. AE, IWTW, and AA: Supervised the study and revised the manuscript. MM, DD, and HS: Supervised the sampling processes and validated the research methods. All authors have read, reviewed, and approved the final manuscript.
